# Uncovering temperature‐dependent extracellular vesicle secretion in breast cancer

**DOI:** 10.1002/jev2.12049

**Published:** 2020-12-31

**Authors:** Kurataka Otsuka, Yusuke Yamamoto, Takahiro Ochiya

**Affiliations:** ^1^ Division of Molecular and Cellular Medicine National Cancer Center Research Institute 5‐1‐1, Tsukiji Chuo‐ku Tokyo Japan; ^2^ R&D Division Kewpie Corporation Sengawa Kewport 2‐5‐7, Sengawa‐cho Chofu‐shi Tokyo Japan; ^3^ Division of Molecular and Cellular Medicine Institute of Medical Science Tokyo Medical University 6‐7‐1, Nishishinjyuku Shinjuku‐ku Tokyo Japan

**Keywords:** breast cancer, cell proliferation, extracellular vesicle, malignancy, temperature

## Abstract

In all living things, temperature is a key factor to maintain function and survive. Animals and plants need to adapt temperature change with optimizing their behaviour and growth by sensing temperature. Similarly, tumour cells must adapt continuously to fluctuations in external conditions including temperature. To find a better environment, cancer cells promote growth and metastasis, which contributes to tumour malignancy. Pathological studies in breast cancer have implied that temperature is associated with disease progression. However, no clear mechanisms have emerged for how thermal changes affect tumour cells and their gene regulation in tumour development and malignancy. Here we discovered the temperature‐dependent extracellular vesicle (EV) secretion in breast cancer. Cancer cell growth and EV secretion increased in a temperature‐dependent manner, which indicated that temperatures were associated with poor prognosis in breast cancer patients. We also found that *low‐density lipoprotein receptor* (*LDLR*), a responsible gene for temperature‐dependent EV secretion, was upregulated with the increase in temperature. Consistent with our results, *LDLR* gene has been characterized and identified as a key factor for malignancy in a wide range of cancers. Our findings shed new light on tumour aggressiveness and therapeutic strategies for breast cancer, especially regarding EV formation and secretion, thus providing a new relationship between cancer and EV biology in the light of temperature.

## 1 INTRODUCTION

Temperature is a key factor for the physiology, development, and behaviour in every organism. For example, in animals, the sex ratio is determined in a temperature‐dependent manner in reptiles (Bull & Vogt, 1979; Ferguson & Joanen, 1982; Warner & Shine, 2008). In plants, the appropriate timing of reproduction (flowering) is controlled in response to temperature (Kumar et al., 2012; Posé et al., 2013). A recent study has shown that temperature affects approximately 30% of the genes expressed in rice (*Oryza sativa*) leaves in a paddy field, and that complex transcriptome changes can be expected by a linear model incorporating meteorological data (Nagano et al., 2012). In addition, plants can adapt to temperature changes to optimize their growth and reproduction by sensing a difference as little as 1°C. In *Arabidopsis*, nucleosomes containing the histone variant H2A.Z centrally act as a thermosensor to sustain gene expression at fluctuating temperature conditions (Kumar & Wigge, 2010). The mutant plants showed a strong thermal induction of flowering and a constitutive high‐temperature transcriptome. Thus, the robust regulation of gene expression against thermal changes is fundamentally necessary in a series of developmental steps to maintain cellular functions.

In humans, tissue and body temperatures are relatively controlled at normal conditions but can be dysregulated when suffering from a disease. In patients suffering from depression, the mean 24‐h body temperature is higher than that in patients in the recovery state or healthy controls (Szuba et al., 1997). In non‐Hodgkin lymphoma, highly malignant tumour cells have higher temperatures, and heat production rates in tumour cells are correlated with poor survival of the patients (Monti et al., 1986). Intriguingly, also in breast cancer, that is the most frequently diagnosed cancer and the leading cause of cancer death among females worldwide, a temperature rise in the tumour area could be recorded, as compared to normal tissue, the maximum difference of which is 3.5°C (Bray et al., 2018; Lawson & Chughtai, 1963). Breast cancer patients with relatively hot tumours had significantly shorter disease‐free and disease‐specific survival than those with cold tumours (Ohsumi et al., 2002). Although pathological studies have indicated that temperature is associated with disease progression and cellular functions in cancer, no clear mechanisms have emerged for how tumour cells react to changes and how thermal conditions affect gene regulation during tumour development and malignancy.

To adapt to changing circumstances, cells communicate and exchange information via diffusible factors, such as cytokines, chemokines, growth factors and hormones. Moreover, it has been increasingly recognized that extracellular vesicles (EVs) act as important mediators of cell‐to‐cell communication, which are secreted from various cell types, including cancer cells, and range in size from 50 to 150 nm in diameter (Dragovic et al., 2011; Valadi et al., 2007; Van Der Pol et al., 2010). In particular, EVs from tumour cells induce angiogenesis, modulate the immune system, and affect the cellular functions of both surrounding and distant cells to support cancer cell growth and pre‐metastatic niche formation (Becker et al., 2016; Costa‐Silva et al., 2015; Hoshino et al., 2015; Peinado et al., 2012). Despite the importance of its clinical application in cancer therapy, yet little is known about the molecular mechanisms between EVs and thermal changes in tumour microenvironments. In this research, we investigated the thermal regulation of breast cancer cell proliferation and gene expression, compared to those of normal breast epithelium cells. By analyzing the altered growth regulation and transcriptome in response to temperature changes, we discovered the temperature‐dependent secretion of EVs and identified the responsible gene, *low*‐*density lipoprotein receptor* (*LDLR*), the expression of which is also thermally controlled.

## 2 MATERIALS AND METHODS

### 2.1 Cell culture

MDA‐MB‐231‐luc‐D3H2LN (Xenogen) and MCF‐7 (ATCC) cells were cultured in RPMI 1640 medium supplemented with 10% heat‐inactivated foetal bovine serum (FBS) and 1% antibiotic‐antimycotic (Thermo Fisher Scientific, Waltham, MA, USA) at 35°C, 37°C, or 39°C in 5% CO_2_. MCF10A cells were cultured in MEBM Mammary Epithelial Cell Basal Medium supplemented with SingleQuots (bovine purified extract, hydrocortisone, human recombinant epidermal growth factor, insulin and amphotericin‐B) (Lonza, Basel, Switzerland) at 35°C, 37°C, or 39°C in 5% CO_2_. Observations were made using an inverted microscope (CKX53; Olympus, Tokyo, Japan).

### 2.2 Cell proliferation assay

Five thousand cells per well were seeded into 96‐well plates and cultured for 0, 24, 48, or 72 h. Cell viability was measured at each time point using the Cell Counting Kit‐8 (Dojindo Laboratories, Kumamoto, Japan). The plates were incubated for 2 h after adding Cell Counting Kit‐8 solution. The absorbance at 450 nm was measured with a Synergy H4 microplate reader (Biotek, Winooski, VT, USA). The experiments were repeated independently at least four times.

### 2.3 Cell migration and invasion assays

Twenty thousand cells in serum‐free media were placed in the upper chamber of Transwell inserts (8‐μm pore size) for migration assays or Matrigel‐coated Transwell inserts for invasion assays (Corning Life Sciences, Tewksbury, MA, USA). The lower chambers contained RPMI supplemented with 10% FBS. After incubation at 35°C, 37°C, or 39°C for 2 h for migration assay or 15 h for invasion assay, cells on the upper surfaces of the membrane present at the interface were removed using cotton swabs. Migrated or invaded cells at the lower surface of the membrane were fixed and stained with Diff‐Quik (Sysmex, Kobe, Japan). The cells were counted in four randomly selected microscopic fields under 20× magnification. The experiments were repeated independently three times.

### 2.4 Microarray analyses

For mRNA microarray analysis, total RNAs (50 ng) were extracted with QIAzol reagent (QIAGEN, Hilden, Germany) from MDA‐MB‐231‐luc‐D3H2LN cells cultured at 35°C, 37°C, or 39°C for 2 days and purified using an RNeasy microkit (QIAGEN). SurePrint G3 Human GE 8×60 K arrays (Agilent Technologies) were hybridized with cyanine 3‐labeled cRNA targets prepared from the RNA samples according to the manufacturer's instructions. The experiments were repeated independently three times. The arrays were analyzed with a Microarray Scanner (Agilent Technologies, Santa Clara, CA, USA). Gene expression levels were calculated using Feature Extraction version 10.7.3.1 software (Agilent Technologies). The normalized and log‐transformed intensity values were analyzed with the GeneSpring GX 7.3.1 (Agilent Technologies). The intensity values were log_2_‐transformed and imported into the Partek Genomics Suite 6.6 (Partek, Inc., Chesterfield, MO, USA). One‐way analysis of variance (ANOVA) was performed to identify differentially expressed genes. Unsupervised clustering and heat map generation were performed with sorted data sets by Euclidean distance based on average linkage clustering, and principal component analysis (PCA) mapping was conducted using all probe sets by Partek Genomics Suite 6.6. Gene set enrichment analysis (GSEA, http://software.broadinstitute.org/gsea/index.jsp) was performed to compare samples cultured at 35°C, 37°C, and 39°C.

### 2.5 EV isolation and analysis

One million cells per dish were plated and incubated for 24 h. The cultured cells were washed with PBS, and the culture medium was replaced with Advanced RPMI 1640 medium (Thermo Fisher Scientific) supplemented with 1% (v/v) antibiotic‐antimycotic and 2 mM L‐glutamine (Thermo Fisher Scientific). After incubation for 24 h, the conditioned medium was collected and centrifuged at 2000 *g* for 10 min at 4°C and the supernatant was filtered through a 0.22 mm filter (Millipore, Billerica, MA, USA). The filtered conditioned medium was transferred to Ultra‐Clear centrifuge tubes (14 × 89 mm; Beckman Coulter, Brea, CA, USA) and centrifuged at 35,000 rpm (110,000 *g*; adjust *k*‐factor = 170.16; and acceleration/deceleration  =  0/0) for 70 min at 4°C in Optima XE‐90 (Beckman Coulter) using a SW41Ti rotor (Beckman Coulter). The pellets were then washed with PBS, centrifuged at 110,000*g* for 70 min at 4°C in Optima XE‐90 using a SW41Ti rotor, and resuspended in PBS. To determine the size distribution of the EVs, the samples were diluted by 10‐fold with PBS for analysis. The size distribution and particle concentration were measured using the Nanosight system LM10 with NTA2.3 Analytical software (NanoSight, Malvern Instruments, Malvern, UK). The measurement conditions were as follows: detection threshold = 7; camera level = 12, frames per second = 25, and detection time = 60 s. The system focuses a laser beam through a suspension of particles. The particles are visualized by light scattering using a conventional optical microscope perpendicularly aligned to the beam axis, which collects light scattered from each particle in the field of view. The Brownian motion of each particle was tracked between frames to calculate its size using the Stokes–Einstein equation. The experiments were repeated independently at least four times. The experiments were performed according to the Minimal Information for Study of Extracellular Vesicles 2018 (MISEV2018) guidelines (Théry et al., 2018). We have submitted all relevant data of our experiments to the EV‐TRACK knowledgebase (EV‐TRACK ID: EV200124) (Van Deun et al., 2017).

### 2.6 Quantitative real‐time PCR analysis

Total RNA and miRNA were extracted from cultured cells using the QIAzol and miRNeasy Mini Kit (QIAGEN) according to the manufacturer's protocol. cDNA was generated using the PrimeScript RT reagent Kit (TaKaRa, Shiga, Japan) and TaqMan MicroRNA Reverse Transcript Kit (Applied Biosystems, Foster City, CA, USA). TaqMan probes (TaqMan Gene Expression Assays and TaqMan MicroRNA Assays) were purchased from Applied Biosystems. qRT‐PCR was performed with the TaqMan probes using Premix Ex Taq (Probe qPCR) (TaKaRa) and TaqMan Universal PCR Master Mix, no AmpErase UNG (Thermo Fisher Scientific) on the StepOne Real‐Time PCR system (Applied Biosystems). The experiments were repeated independently at least three times. Expression levels of the gene of interest were normalized to the expression of *GAPDH*.

### 2.7 Cryogenic electron microscopy

EVs were collected from MDA‐MB‐231‐luc‐D3H2LN cells cultured at 35°C, 37°C, or 39°C for 1 day as described above. Each sample solution (2.5 μl) was applied to a Quantifoil R1.2/1.3 Mo grid (Quantifoil Micro Tools GmbH, Jena, Germany) that had previously been glow‐discharged, and quickly frozen in a liquid ethane using Vitrobot Mark IV (Thermo Fisher Scientific) at 95% humidity and 4°C. Frozen grids were imaged with a JEM‐2200FS electron microscope operating at 200 kV accelerating voltage equipped with an omega‐type energy filter and field emission electron source (JEOL, Tokyo, Japan). Images were recorded on a DE‐20 direct detector (Direct Electron LP, San Diego, CA, USA) at a nominal magnification of 25K.

### 2.8 Transmission electron microscopy

MDA‐MB‐231‐luc‐D3H2LN cells were cultured at 35°C, 37°C, or 39°C for 1 day and fixed with 2% paraformaldehyde and 2% glutaraldehyde (GA) in 0.1 M phosphate buffer (pH 7.4) at room temperature (RT). The samples were incubated at 4°C for 30 min, and fixed with 2% GA in 0.1 M phosphate buffer at 4°C overnight. They were washed three times with 0.1 M phosphate buffer and post‐fixed with 2% osmium tetroxide in 0.1 M phosphate buffer at 4°C for 1 h. The samples were dehydrated in graded ethanol solutions (50%, 70%, 90%, and 100%) as follows: 50% and 70% for 5 min each at 4°C, 90% for 5 min at RT, and three changes of 100% for 5 min each at RT. The samples were embedded in Quetol‐812 (Nisshin EM, Tokyo, Japan) and polymerized at 60°C for 48 h. The polymerized resins were sectioned at 70 nm using an ultramicrotome (Ultracut‐UCT; Leica, Wetzlar, Germany). The sections were mounted on copper grids, stained with 2% uranyl acetate at RT for 15 min, washed with distilled water, and stained with Lead stain solution (Sigma‐Aldrich, St. Louis, MO, USA) at RT for 3 min. The grids were observed using transmission electron microscopy (JEM‐1400Plus; JEOL) at an acceleration voltage of 100 kV. Digital images were acquired with a CCD camera (EM‐14830RUBY2; JEOL).

### 2.9 Western blotting

EV samples were denatured in 4× sample buffer solution with 2‐mercaptoethanol (FUJIFILM Wako Pure Chemical Corporation, Osaka, Japan) for ALIX and APOA1, and without 2‐mercaptoethanol (FUJIFILM Wako Pure Chemical Corporation) for CD9 and CD63 at 95°C for 5 min. Total protein (500 ng) was loaded into each lane, separated using SDS‐polyacrylamide gel electrophoresis (4%–20% Mini‐PROTEAN TGX Gel; Bio‐Rad, Hercules, CA, USA), and transferred to a polyvinylidene fluoride (PVDF) membrane (Millipore). The membranes were incubated at RT for 1 h with Blocking One (Nacalai Tesque, Kyoto, Japan). They were then incubated with primary antibodies, followed by secondary antibodies. The blots were developed by using immunoStar LD (FUJIFILM Wako Pure Chemical Corporation). Images were acquired using a FUSION SOLO 7S chemilumincescence imaging system (Vilber Lourmat, Marne‐la‐Vallée, France). The experiments were repeated independently three times. Antibodies against ALIX (Millipore, ABC40), APOA1 (R&D Systems, Minneapolis, MN, USA, AF3664), CD9 (Cosmo Bio, Beijing, China, 12A12), and CD63 (Cosmo Bio, 8A12) were used. We have confirmed that the antibodies are suitable for immunoblotting. The secondary antibodies were horseradish peroxidase (HRP)‐conjugated sheep anti‐mouse IgG (GE Healthcare Life Sciences, Little Chalfont, UK, NA931V) and HRP‐conjugated donkey anti‐rabbit IgG (GE Healthcare Life Sciences, NA934V).

### 2.10 Transient transfection assays

For siRNA transfection, pre‐designed siRNAs targeting *LDLR* mRNA (ID: s224006 and s224007) were purchased from Applied Biosystems. The *Silencer* Select Negative Control #1 siRNA (Applied Biosystems) was used as a negative control. Transfection was performed using DharmaFECT transfection reagent (Thermo Fisher Scientific) according to the manufacturer's protocol. A total of 25 nM siRNA or miRNA was used for each transfection. The experiments were repeated independently at least three times.

### 2.11 Statistical analysis

The data are presented as the means ± standard error of the mean (SEM) of at least three independent experiments. Statistical analyses on data containing more than two groups were performed using one‐way ANOVA followed by Tukey's honestly significance difference test to account for multiple comparisons. *P* < 0.05 was considered as statistically significant.

## 3 RESULTS

### 3.1 Effects of temperature changes on aggressive breast cancer cell phenotype

**FIGURE 1:**
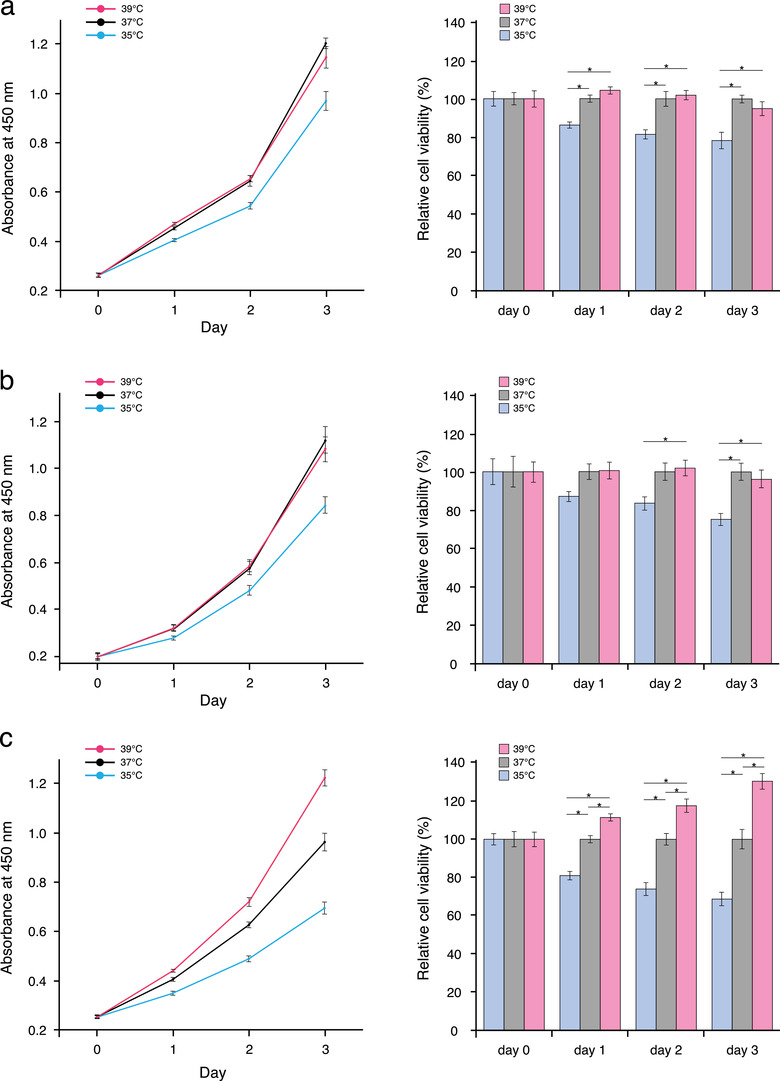
Effects of temperature on the proliferation of breast cancer (a) Effect of temperature on the proliferation of normal human mammary epithelial cells (MCF10A cells) (mean ± SEM). Left: cell viability at 0, 1, 2, and 3 days after changing the culture temperature. Right: cell proliferation normalized to that at 37°C on each day (*P < 0.05). Number of data points per sample was five. (b) Effect of temperature on the proliferation of luminal‐type breast cancer cells (MCF‐7 cells) (mean ± SEM). Left: cell viability at 0, 1, 2, and 3 days after changing the culture temperature. Right: cell proliferation normalized to that at 37°C on each day (*P < 0.05). Number of data points per sample was five. (c) Effect of temperature on the proliferation of basal‐type breast cancer cells (MDA‐MB‐231‐luc‐D3H2LN cells) (mean ± SEM). Left: cell viability at 0, 1, 2, and 3 days after changing the culture temperature. Right: cell proliferation normalized to that at 37°C on each day (*P < 0.05). Number of data points per sample was five

**FIGURE 2:**
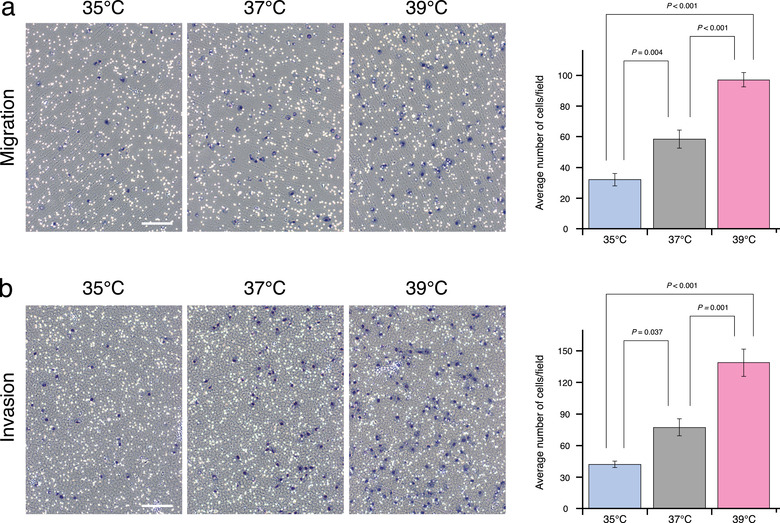
Effects of temperature on the migration and invasion of aggressive breast cancer cells. (a) Migration of MDA‐MB‐231 cells. Left: migrated cells incubated at 35°C, 37°C, or 39°C. Scale bar represents 100 μm. Right: average number of migrated cells/field at each temperature (mean ± SEM). Number of data points per sample was six. (b) Invasion of MDA‐MB‐231 cells. Left: invaded cells incubated at 35°C, 37°C, or 39°C. Scale bar represents 100 μm. Right: average number of invaded cells/field at each temperature (mean ± SEM). Number of data points per sample was six

We hypothesized that in breast cancer, tumour aggressiveness is conceptually defined by the combination of: (1) temperature‐dependent cell proliferation and (2) temperature‐dependent EV regulation. To test this hypothesis, we first examined the characteristics of cell proliferation in response to temperature changes in normal human mammary epithelial cells (MCF10A cells), luminal‐type (MCF‐7 cells), and basal‐type breast cancer cells (MDA‐MB‐231‐luc‐D3H2LN cells). When we exposed the cells to different temperatures (35°C as cool, 37°C as normal, or 39°C as warm temperatures), morphological changes were not observed in each cell lines cultured at each temperature (Supplementary Figure S1–S3). Interestingly, the proliferation of epithelial cells and luminal‐type cancer cells at 39°C showed a similar trend as cultured obtained at 37°C (Figure 1(a, b) and Supplementary Figure S4). This suggested that there is a mechanism to prevent excess cell growth at higher temperatures in normal human mammary epithelial and luminal‐type cancer cells. In contrast, the proliferation of triple‐negative breast cancer cells was promoted in a temperature‐dependent manner, suggesting that cell growth regulation in response to temperature was defective in aggressive cancer cells (Figure 1(c) and Supplementary Figure S4–S6). In addition, temperature also induced MDA‐MB‐231 cell migration and invasion (Figure 2). In the light of the previous pathological studies, these results suggested that temperature is a key factor for controlling cell proliferation, migration, and invasion in breast cancer, and thus prognosis in patients.

### 3.2 Comprehensive transcriptome analysis of temperature responses in breast cancer

**FIGURE 3:**
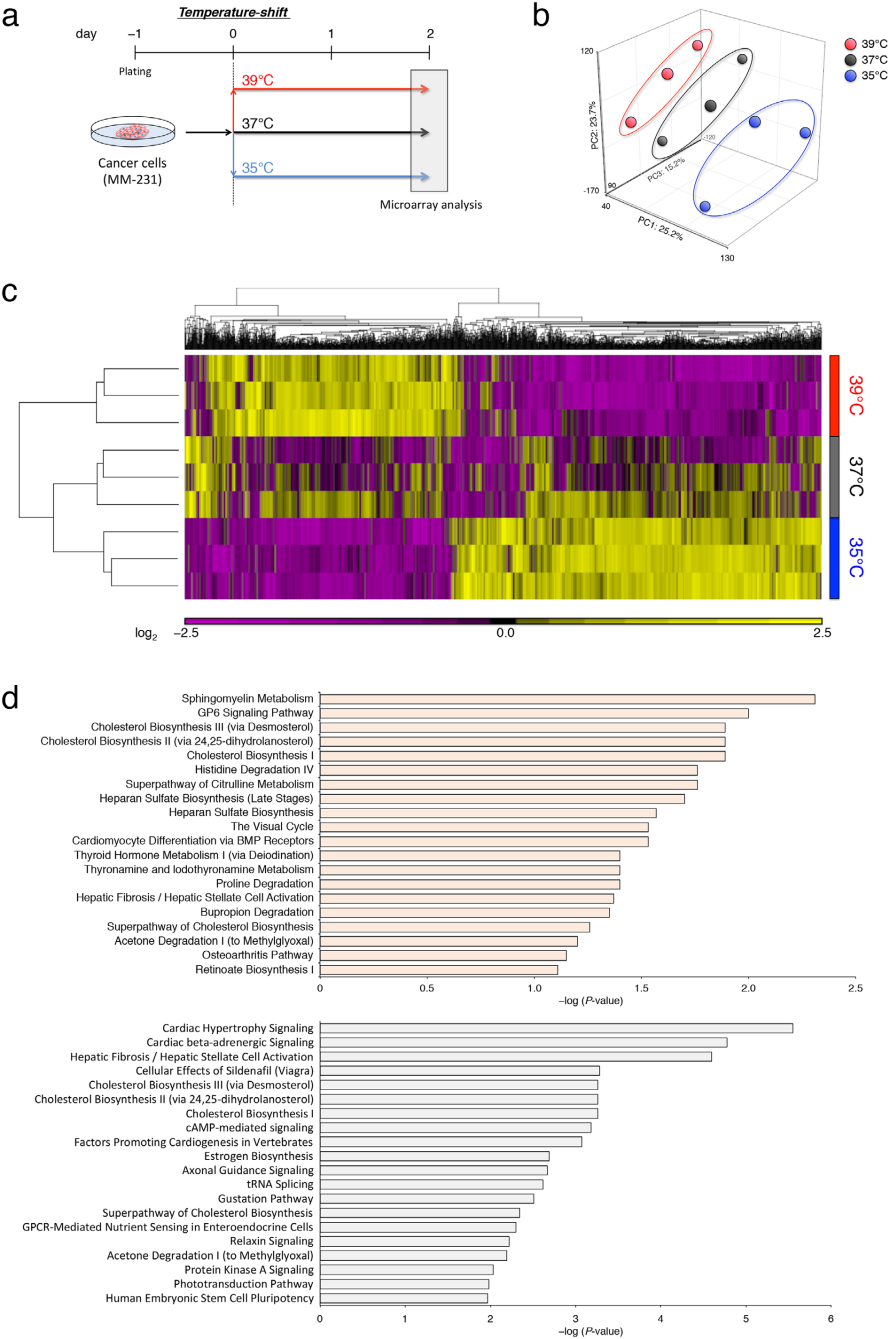
Effects of temperature on the transcriptome profiles in breast cancer. (a) Schematic diagram of temperature‐shift experiments for microarray analysis. (b) Principle component analysis of microarray samples separated by groups. Breast cancer cells were cultured for 2 days at 35°C (blue), 37°C (black), and 39°C (red). Number of data points per group was three. (c) Heat map showing genes that were significantly downregulated or upregulated by temperature changes (*P* < 0.05). (d) Canonical pathways identified by Ingenuity Pathway Analysis for differentially expressed genes in microarray data. The top 20 pathways were obtained by analyzing the microarray data from breast cancer cells cultured at 37°C versus that of 35°C (upper) and from breast cancer cells cultured at 39°C versus that of 35°C (bottom). The horizontal axis in the graphs indicates statistical significance

To study the characteristics of breast cancer cells in response to temperature changes, we shifted the culture temperature from 37°C to 35°C, 37°C, or 39°C and examined the effects of these treatment on gene expression in MDA‐MB‐231 cells (Figure 3(a)). The microarray analysis clearly revealed transcriptome changes between cancer cells cultured at different temperatures (Figure 3(b)). In addition, we observed approximately 2600 genes whose expression levels were thermally controlled (Figure 3(c)). To explore the biological implications of thermal adaptation in breast cancer, we investigated the functional categories of differentially expressed genes in response to temperature. Surprisingly, in agreement with our hypothesis, pathway analysis revealed that sphingomyelin metabolism was identified as the top affected canonical pathway, including *sphingomyelin phosphodiesterase 3* (*SMPD3*), which regulates EV secretion (Figure 3(d) and Supplementary Figure S7) (Kosaka et al., 2010). These data suggested that temperature conditions could affect EV secretion besides cancer cell growth in breast cancer, which led us to further investigate whether EV secretion from tumour cells was controlled by temperature changes.

### 3.3 Temperature‐dependent regulation of EV secretion in breast cancer

**FIGURE 4:**
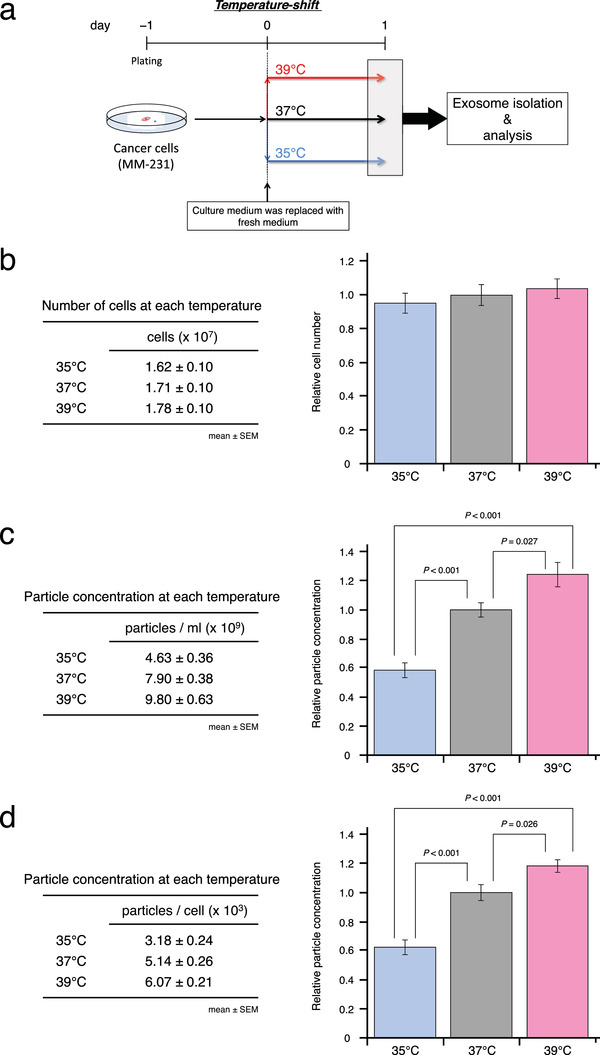
Effects of temperature on EV secretion in breast cancer. (a) Schematic diagram of temperature‐shift experiments for EV isolation and analysis. (b) Number of cells at the time of EV isolation. Breast cancer cells were cultured at 35°C, 37°C, or 39°C for only 1 day after the temperature‐shift. Left: number of cells at each temperature. Right: cell numbers normalized to that of 37°C. Number of data points per sample was eight. (c) Particle concentration of EVs from breast cancer cells cultured at 35°C, 37°C, or 39°C (mean ± SEM). Left: particle concentration of EVs (particles/ml). Right: particle concentration of EVs normalized to that of 37°C. Number of data points per sample was eight. (d) Particle concentration of EVs from breast cancer cells cultured at 35°C, 37°C, or 39°C (mean ± SEM). Left: particle concentration of EVs (particles/cell). Right: particle concentration of EVs normalized to that of 37°C. Number of data points per sample was eight

For the analysis of EVs released from breast cancer cells, we cultured MDA‐MB‐231 cells for only 1 day after the temperature‐shift, in order to avoid the saturation of EVs in the culture medium and to start with almost the same number of cultured living cells at each temperature condition (Figure 4(a)). When we collected cell culture supernatants, cell numbers were counted and calculated in each condition, and then we confirmed that the numbers were not different between the three temperature conditions (Figure 4(b)). By using standard EV purification techniques (Théry et al., 2018), EVs were purified from the cell supernatants by ultracentrifugation. When compared to each temperature condition, particle concentrations were significantly changed in a temperature‐dependent manner (Figure 4(c)). Even after normalizing the number of cells, the temperature‐dependency of EV secretion was observed (Figure 4(d)). Notably, the ratio of the number of secreted EVs was higher when switching from 35°C to 37°C than from 37°C to 39°C. These results indicated that EV secretion from breast cancer cells increased with an increase in temperature.

### 3.4 Temperature effects on physical characteristics of EVs in breast cancer cells

**FIGURE 5:**
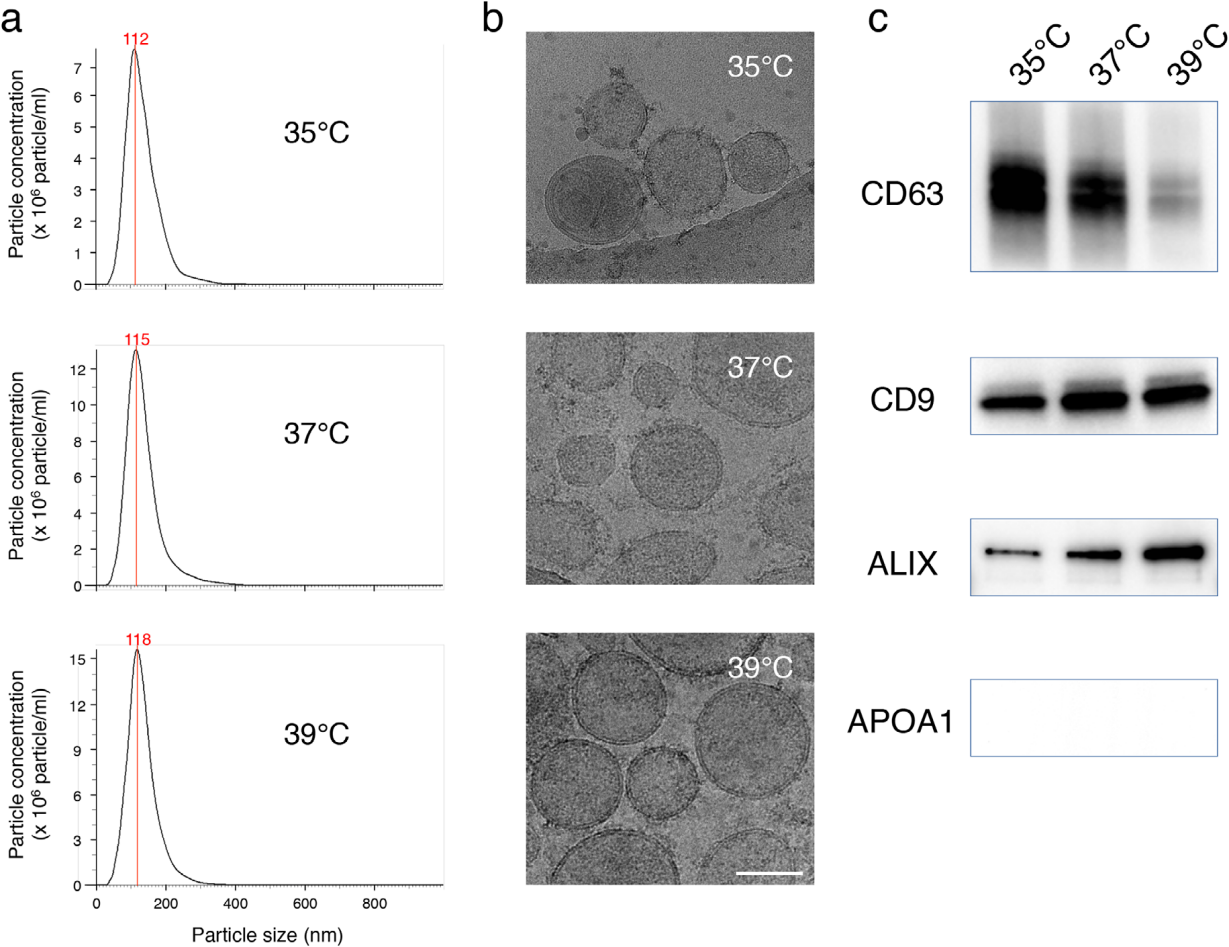
Characterization of EVs from breast cancer cells cultured at each temperature. (a) Size distribution of EVs from cancer cells cultured at different temperatures. The sizes of EVs from cancer cells cultured at 35°C, 37°C, or 39°C were analyzed using a NanoSight nanoparticle tracking system. The vertical and the horizontal axes in the graphs indicate the number of EV particles (×10^6^)/ml and particle size (nm), respectively. Numbers (red colour) shown on graphs are the average sizes of the EVs. (b) Electron microscopy analysis of EVs. Representative images of EVs from MDA‐MB‐231 cells cultured at 35°C, 37°C, or 39°C. Scale bar represents 100 nm. (c) Western blot for CD63, CD9, ALIX, and APOA1 of EVs from MDA‐MB‐231 cells cultured at 35°C, 37°C, or 39°C

The sizes of secreted EVs, determined by nanoparticle tracking analysis, were almost the same among the three groups (35°C, 37°C, and 39°C) and categorized as small EVs based on their size (Figure 5(a)) (Théry et al., 2018). In addition, when the EVs isolated from MDA‐MB‐231 cells at each temperature condition were observed using cryo‐electron microscopy, the vesicular structures did not differ among the three temperature conditions (Figure 5(b) and Supplementary Figure S8). Next, the protein composition of the EVs was analyzed with three categories of EV markers by western blotting. The common EV markers CD9, CD63 (category 1), and ALIX (category 2) were confirmed from the EVs from breast cancer cells exposed to each temperature, supporting the presence of EVs (Figure 5(c) and Supplementary Figure S9). The category 3 marker apolipoprotein A1 (APOA1) was not detected, confirming the purity of the EVs (Figure 5(c)). Interestingly, CD63 expression was decreased in a temperature‐dependent manner. On the other hand, ALIX was increased in a temperature‐dependent fashion. CD9 was slightly induced by temperature change. The data showed that the protein composition of EVs was strongly affected by the temperature condition, suggesting that temperature would change the physical characteristics of EVs.

### 3.5 Identification of the responsible gene for temperature‐dependent EV secretion in breast cancer

**FIGURE 6:**
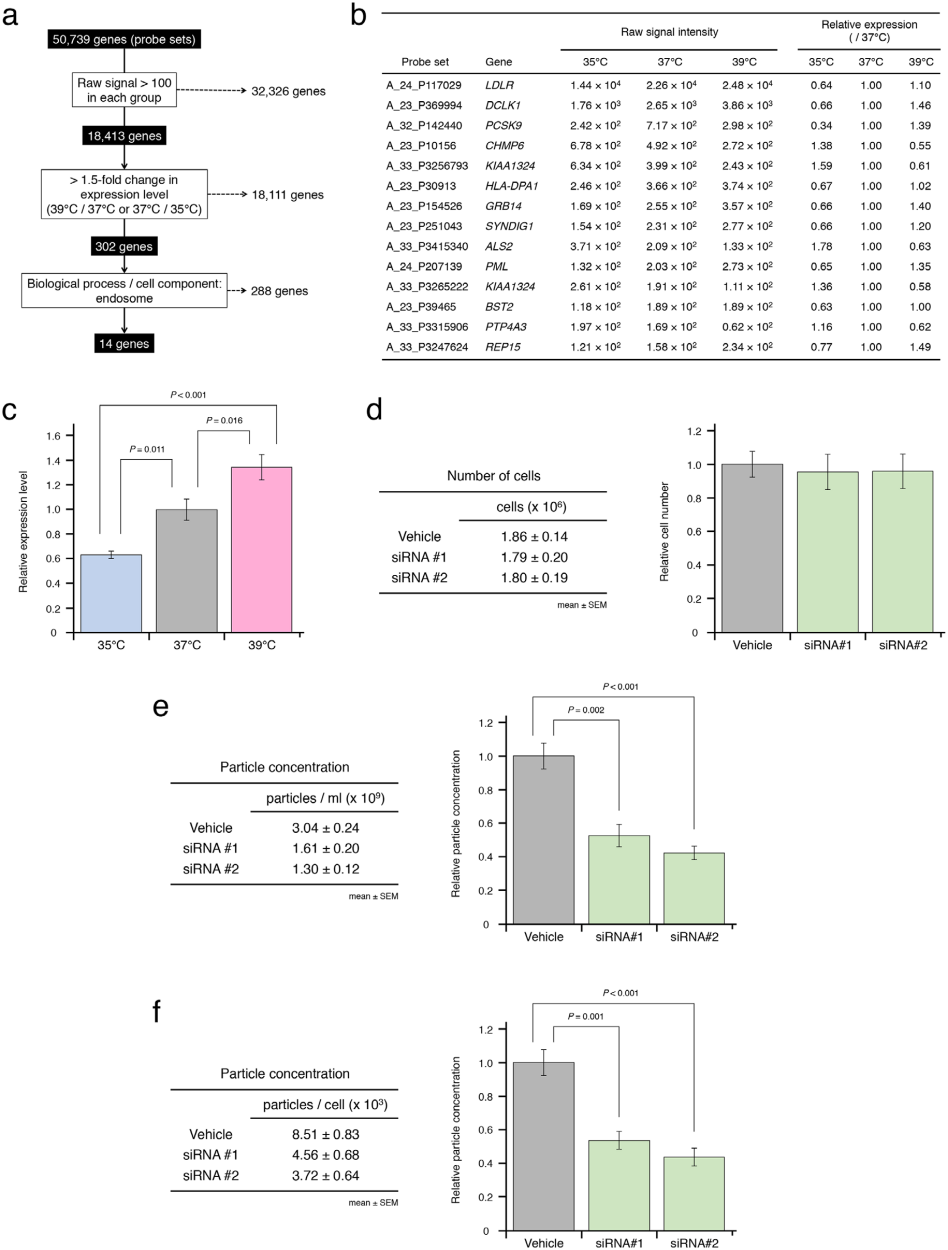
Effects of temperature on EV secretion in breast cancer. (a) Flow chart of selected candidate genes involved in EV secretion. (b) Candidate genes responsible for temperature‐dependent EV secretion in breast cancer. The list of genes was obtained from microarray analysis. The table shows raw signal intensity and relative expression levels that were normalized to that at 37°C. (c) Effect of temperature on *LDLR* expression (mean ± SEM). The expression levels were confirmed by qRT‐PCR and normalized to the expression of *GAPDH*. Number of data points per sample was six. (d) Number of cells at the time of EV isolation. *LDLR* knockdown cells were cultured at 37°C for only 1 day after replacement of fresh medium. Left: number of cells at each temperature. Right: cell numbers normalized to that of vehicle treatment. Number of data points per sample was four. (e) Particle concentration of EVs from *LDLR* knockdown cells cultured at 37°C (mean ± SEM). Left: particle concentration of EVs (particles/ml). Right: particle concentration of EVs normalized to that of vehicle treatment. Number of data points per sample was four. (f) Particle concentration of EVs from *LDLR* knockdown cells cultured at 37°C (mean ± SEM). Left: particle concentration of EVs (particles/cell). Right: particle concentration of EVs normalized to that of vehicle treatment. Number of data points per sample was four

To elucidate the molecular mechanisms underlying this thermal regulation of EV secretion in breast cancer, we analyzed the differential transcriptional expression of genes during temperature changes (35°C, 37°C, or 39°C) in cancer cells (Figure 3(a–c)), and narrowed down the list of potential responsible genes according to the criteria shown in Figure 4A. Potential involvement in endosome was considered one of the criteria, because EVs are formed by the inward budding of endosomal membranes during maturation of multivesicular bodies (MVBs) and released upon fusion of MVBs with the cell surface (Van Niel et al., 2018). Therefore, we identified 14 putative responsible genes for temperature‐dependent EV secretion (Figure 6(a)). Among those genes, we focused on low‐density lipoprotein receptor (*LDLR*) because of its high expression levels and the existence of several cholesterol‐related pathways that were affected with temperature (Figure 6(b)). qRT‐PCR analysis showed that *LDLR* expression was significantly upregulated by temperature increase and correlated with the number of secreted EVs in response to temperature changes (Figure 4(c, d) and Figure 6(c)).

Next, we knocked down *LDLR* gene expression in order to determine its functional effects on EV secretion. Cancer cells were transfected with siRNA, followed by replacement of fresh medium, and cultured for only 1 day after transfection. We confirmed that the cell numbers did not decrease in *LDLR*‐knockdown cells cultured for 1 day when the cell culture supernatants were collected (Figure 6(d) and Supplementary Figure S10). Nonetheless, in *LDLR*‐knockdown cells, particle concentrations were significantly decreased, which showed that *LDLR* was linked to EV formation and secretion (Figure 6(e, f) and Supplementary Figure S11).

Recent studies have reported that EVs from cancer cells affect the cellular functions of both surrounding and distant cells to support tumour progression and metastasis. Our results showed that the expression levels of *LDLR* were correlated with the number of secreted EVs by breast cancer cells. Consequently, to evaluate the prognostic value of *LDLR* expression in breast cancer, we investigated the association between *LDLR* expression in breast cancer samples and disease progression (Györffy et al., 2010). Intriguingly, Kaplan–Meier survival analysis demonstrated that patients with high *LDLR* expression experienced shorter overall survival than patients with low *LDLR* expression and that patients with high *LDLR* expression had a significantly shorter distant metastasis‐free survival than patients with low *LDLR* expression (*P* = 0.0037 by log‐rank test; Supplementary Figure S12). These results suggested that breast cancer metastasis could be promoted by increased EV secretion that occurred as a consequence of *LDLR* upregulation.

## 4 DISCUSSION

In all organisms, temperature is critical for survival and evolution. In living cells, temperature is a very important factor for enzyme activity and stability (biological reaction) to maintain the function and structure by producing biomolecules, such as nucleic acids and proteins. In order to adapt to temperature changes, animals generally select an appropriate temperature and move to a better environment. Plants need to optimize their growth and reproduction by sensing temperature and modulating gene expression. In cancer, as is the case with plants, tumour cells must adapt continuously to fluctuations in external conditions, including temperature. To find optimal surroundings, cancer cells promote growth and metastasis, which contributes to tumour malignancy. In breast cancer, some pathological studies have implied that temperature rise in tumours is associated with disease progression. However, no clear mechanisms have emerged on how tumour cells react to changes and how thermal conditions affect gene regulation and EV secretion in tumour development and malignancy. Here we discovered a mechanism of temperature‐dependent EV secretion in breast cancer. Cancer cell growth and EV secretion were promoted at higher temperature, which indicated that temperature was related to poor prognosis patients with breast cancer.

We also found the responsible gene *LDLR*, the expression of which was upregulated by temperature elevation. LDLR is a cell‐surface receptor that regulates cholesterol levels. Many studies have demonstrated increased *LDLR* expression and LDL cholesterol uptake in a variety of tumours, such as leukemia, lung cancer, glioblastoma, breast cancer, and pancreatic cancers (Guillaumond et al., 2015; Guo et al., 2011; Kimbung et al., 2016; Vitols et al., 1990, 1992). Proliferating cancer cells generally require higher uptake of cholesterol with high expression of LDLR because of the need for plasma membrane construction and an extra energy source to sustain growth (Furuya et al., 2016; Haeffner et al., 1984; Vitols et al., 1984;Vitols et al., 1996; Zhou et al., 2017). Cholesterol serves as an essential structural component of the plasma membrane by organizing lipid rafts that are enriched in sphingolipids such as sphingomyelin (Edidin, 2003). In tumours, as lipid rafts possess many types of receptor proteins, cholesterol has an impact on tumour signalling pathways through alterations of signal transduction (Freeman & Solomon, 2004; Patra, 2008; Zhuang et al., 2005). In malignant breast cancer tissues, *LDLR* expression and cholesterol levels were increased, compared to those in benign breast tissues (Antalis et al., 2011). The activation of the Ras/Raf‐1/MEK/ERK pathway was observed in aggressive breast cancer cells, and ERK1/2 activation was correlated with *LDLR* expression levels (Antalis et al., 2011). Both PKC and MEK inhibitors decreased the expression levels of LDLR and reduced cell migration. Another study showed that the anti‐diabetic drug metformin decreased cell migration by inhibiting the expression of cholesterol regulatory genes, including *LDLR* (Sharma et al., 2019). In addition, it has been reported that *LDLR* plays a role in promoting cell proliferation and migration, which contributes to cancer progression (Guillaumond et al., 2015; Jiang et al., 2015; Li et al., 2006; Migita et al., 2009). In other cancers, *LDLR* is also involved in proliferation and migration (Guillaumond et al., 2015; Jiang et al., 2015). These observations have shown that a high expression level of *LDLR* is a key factor in tumour aggressiveness.

Our study showed that in breast cancer, thermally‐regulated *LDLR* plays a role in temperature‐dependent increases in EV secretion. Tumour‐derived EVs have been shown to promote cancer invasion and metastasis by remodelling both surrounding and distant tissues (Hoshino et al., 2015; Kosaka et al., 2013; Tominaga et al., 2015; Zhou et al., 2014). Consistent with our results regarding the relationship between *LDLR* and EVs, *LDLR* is highly expressed in metastatic breast cancer cells as compared to in both nonmetastatic and nontumorigenic cells (Antalis et al., 2011; Sharma et al., 2019). Some groups reported that LDLR was localized to EVs (Demory Beckler et al., 2013; He et al., 2015; Kharaziha et al., 2015; Liang et al., 2013), showing a direct connection between LDLR and EVs. EVs are also involved with cancer recurrence (Chen et al., 2018; Ono et al., 2014). Intriguingly, it was reported that high *LDLR* expression was associated with decreased recurrence‐free survival in breast cancer (Gallagher et al., 2017). By Kaplan–Meier analysis, we determined that patients with high *LDLR* expression had a significantly shorter distant metastasis‐free survival than patients with low *LDLR* expression. Considering that *LDLR* was related to EV secretion, this implied that increased EV production with elevated *LDLR* expression would contribute to the formation of the pre‐metastatic niches. As previously mentioned, many studies have suggested an association between *LDLR* and breast cancer malignancy, but the mechanisms remain to be fully elucidated. Our findings shed new light on tumour aggressiveness and breast cancer progression, particularly regarding EV formation and secretion.

EV production has been shown to be mediated by *SMPD3* (known as *NSMASE2*) and Rab family members (*RAB2B*, *RAB5A*, *RAB9A*, *RAB22A*, *RAB27A*, and *RAB27B*) (Kosaka et al., 2010; Ostenfeld et al., 2014; Ostrowski et al., 2010; Peinado et al., 2012; Wang et al., 2014). Here, we found that *LDLR* is a new player in EV production. To understand the biology of mammalian EVs, it is worth investigating the interaction between cholesterol regulatory genes, including *LDLR* and other genes, such as *SMPD3* and Rab family members. It is currently difficult to evaluate the interaction and the mechanism by which LDLR is involved in EV formation and/or secretion. Because many molecules and sorting machineries are required for the generation and secretion of EVs and microvesicles in different steps, it is complicated to assess the role just by simply disrupting its function or mechanism, and thus more comprehensive research is needed to fully understand the mechanisms. It is also important to cast a spotlight on EV content (nucleic acids, proteins, lipids, and small molecules) because EV cargo contributes to cancer development and has become the focus of study aimed at developing new tools for cancer diagnosis and treatments (El Andaloussi et al., 2013; Hessvik & Llorente, 2018; Maas et al., 2017; Tkach & Théry, 2016; Van Niel et al., 2018). In this study, we have yet to elucidate the difference in the molecular content of EVs in response to temperature change; however, the composition of common specific components, such as CD9, CD63, and ALIX, was changed by temperature, providing insight into the nature of EVs. By transcriptome and proteome analyses, our future investigations will provide additional information regarding EV biology. It is also interesting to study the connection between temperature and EVs in other cancers, because it is still unclear and could be of clinical relevance. Our study indicated the existence of a mechanism for EV production and provided a new perspective on cancer and EV biology, which is dependent on the temperature.

## CONFLICT OF INTEREST

The authors have declared no conflict of interest.

## FUNDING INFORMATION

This work was supported in part by the Japan Agency for Medical Research and Development (AMED) 19cm0106402h0004 (to Takahiro Ochiya) and Takeda Science Foundation (to Kurataka Otsuka and Yusuke Yamamoto).

## AUTHOR CONTRIBUTIONS

Takahiro Ochiya and Kurataka Otsuka directed and supervised the study. Kurataka Otsuka designed and performed most of the experiments, analyzed data, and wrote the manuscript. Yusuke Yamamoto helped with data interpretation for microarray analysis using Ingenuity Pathway Analysis. Kurataka Otsuka and Takahiro Ochiya revised the manuscript. All authors critically reviewed the manuscript and approved the final version of the manuscript.

## Supporting information



Supporting Information

## Data Availability

All data needed to evaluate the conclusions in the paper are present in the paper and the Supplementary Materials. Additional data related to this paper may be requested from the authors.
