# Supplementary material for: Uncovering temperature‐dependent extracellular vesicle secretion in breast cancer
Source: J Extracell Vesicles. 2020 Dec 31;10(2):e12049. doi: 10.1002/jev2.12049 (PMC7775566; doi:10.1002/jev2.12049)
Supplement: Supplementary file 1 — Supporting Information [file JEV2-10-e12049-s001.pdf]

Supplementary Materials

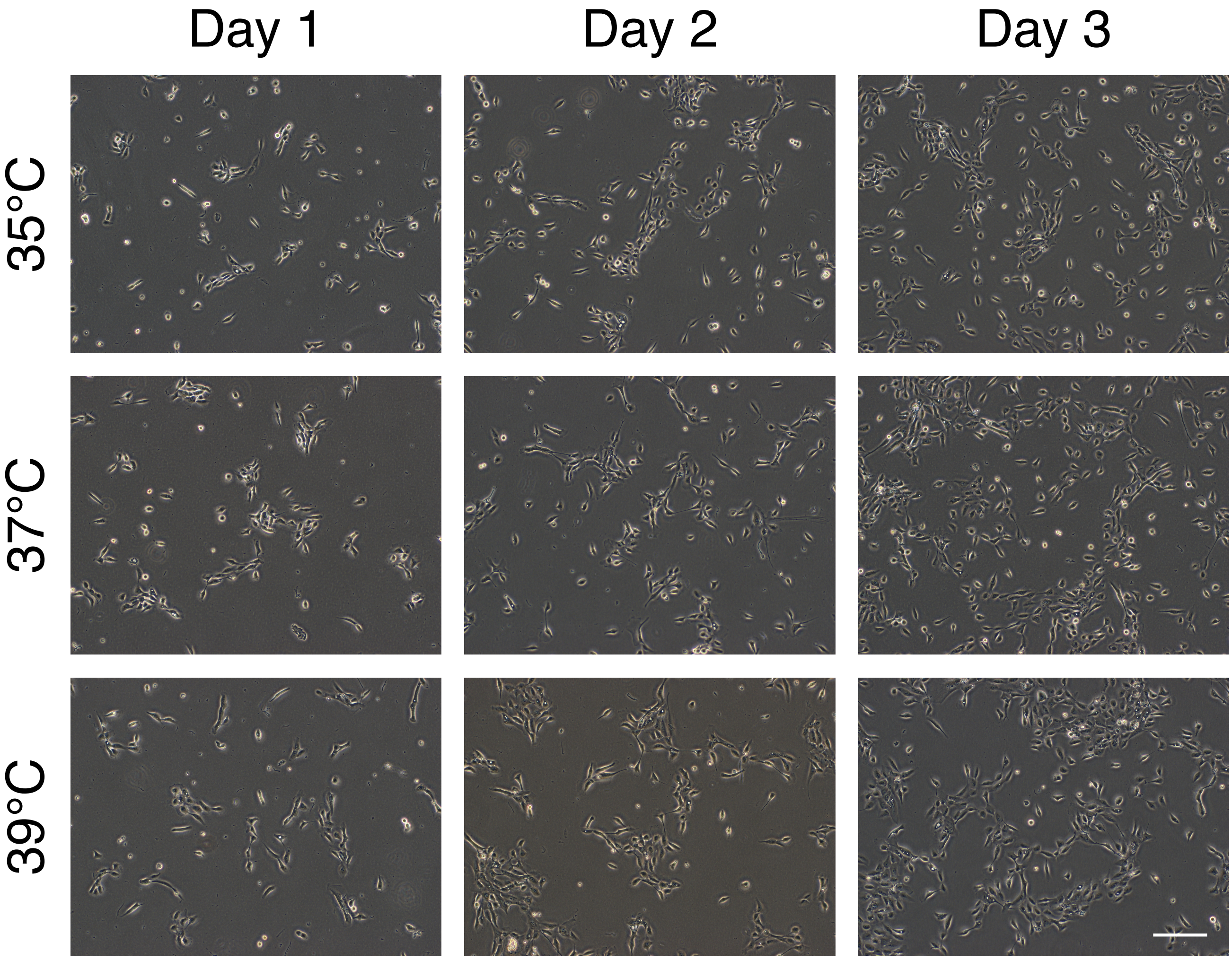

Figure S1. Morphology of MCF10A cells cultured at different temperatures
MCF10A cells were cultured at 35°C, 37°C, or 39°C for 1, 2, and 3 days. Drastic morphological changes were not observed for cells cultured at each temperature. Scale bar represents 200 µm.

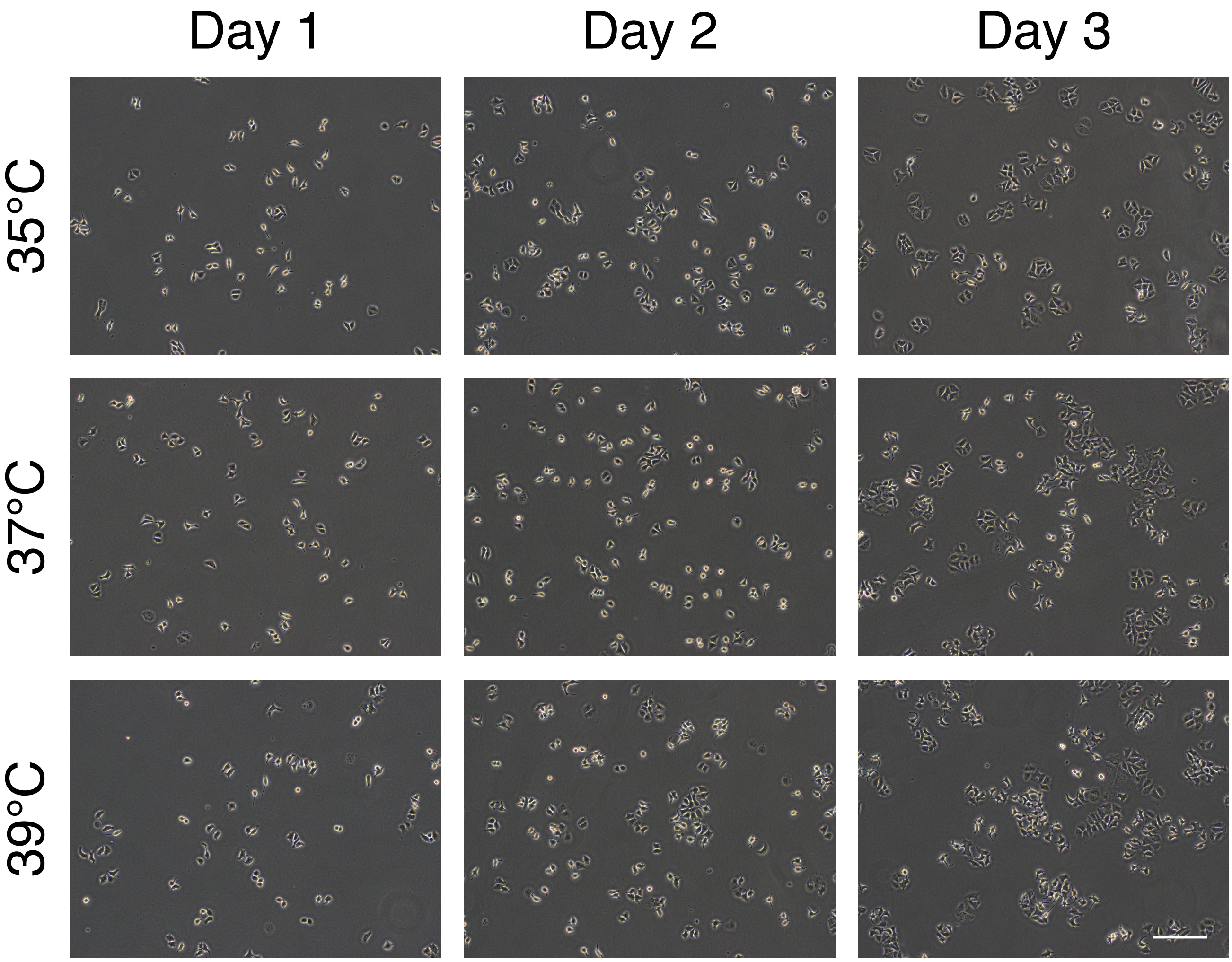

Figure S2. Morphology of MCF-7 cells cultured at different temperatures
MCF-7 cells were cultured at 35°C, 37°C, or 39°C for 1, 2, and 3 days. Drastic morphological changes were not observed for cells cultured at each temperature. Scale bar represents 200 µm.

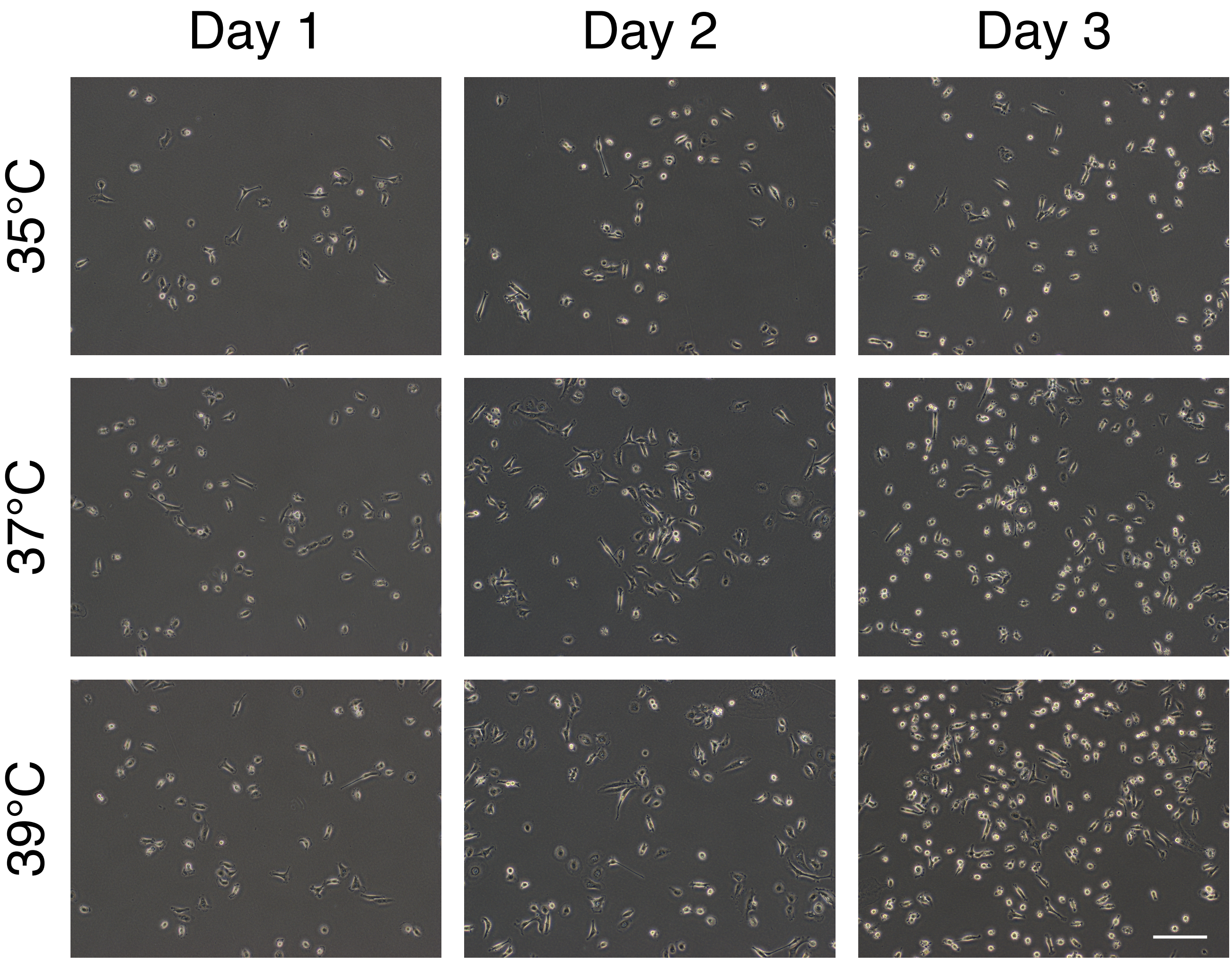

Figure S3. Morphology of MDA-MB-231 cells cultured at different temperatures
MDA-MB-231 cells were cultured at 35°C, 37°C, or 39°C for 1, 2, and 3 days. Drastic morphological changes were not observed for cells cultured at each temperature. Scale bar represents 200 µm.

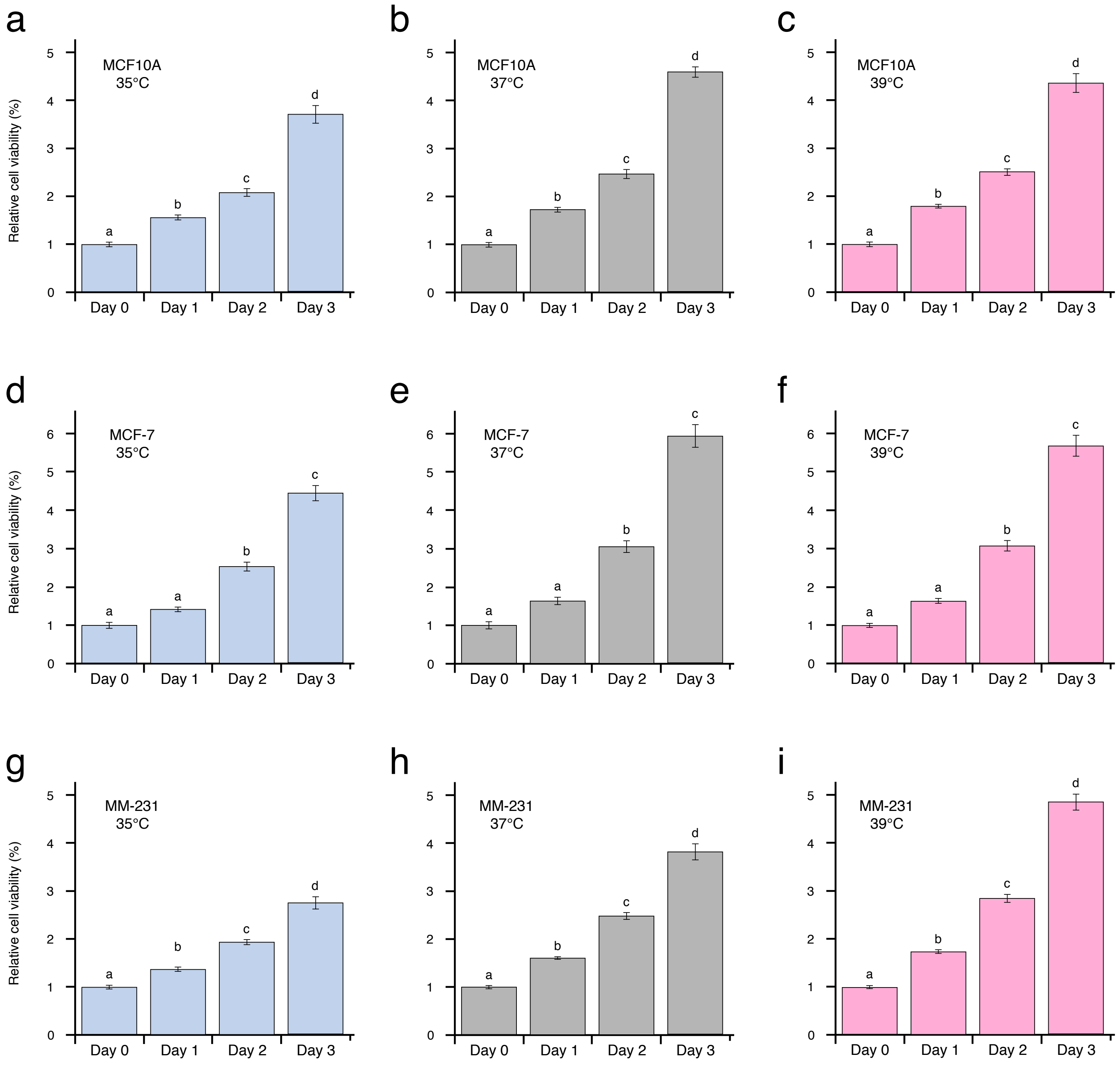

Figure S4. Effects of temperature on cell proliferation

(**a–c**) Effect of temperature on the proliferation of normal human mammary epithelial cells (MCF10A cells) (mean ± SEM).

(**d–f**) Effect of temperature on the proliferation of luminal-type breast cancer cells (MCF-7 cells) (mean ± SEM).

(**g–i**) Effect of temperature on the proliferation of basal-type breast cancer cells (MDA-MB-231-luc-D3H2LN cells) (mean ± SEM).

Cell proliferation was normalized to that of each temperature on day 0. Different letters indicate significant differences at $P < 0.05$. Number of data points per sample was five.

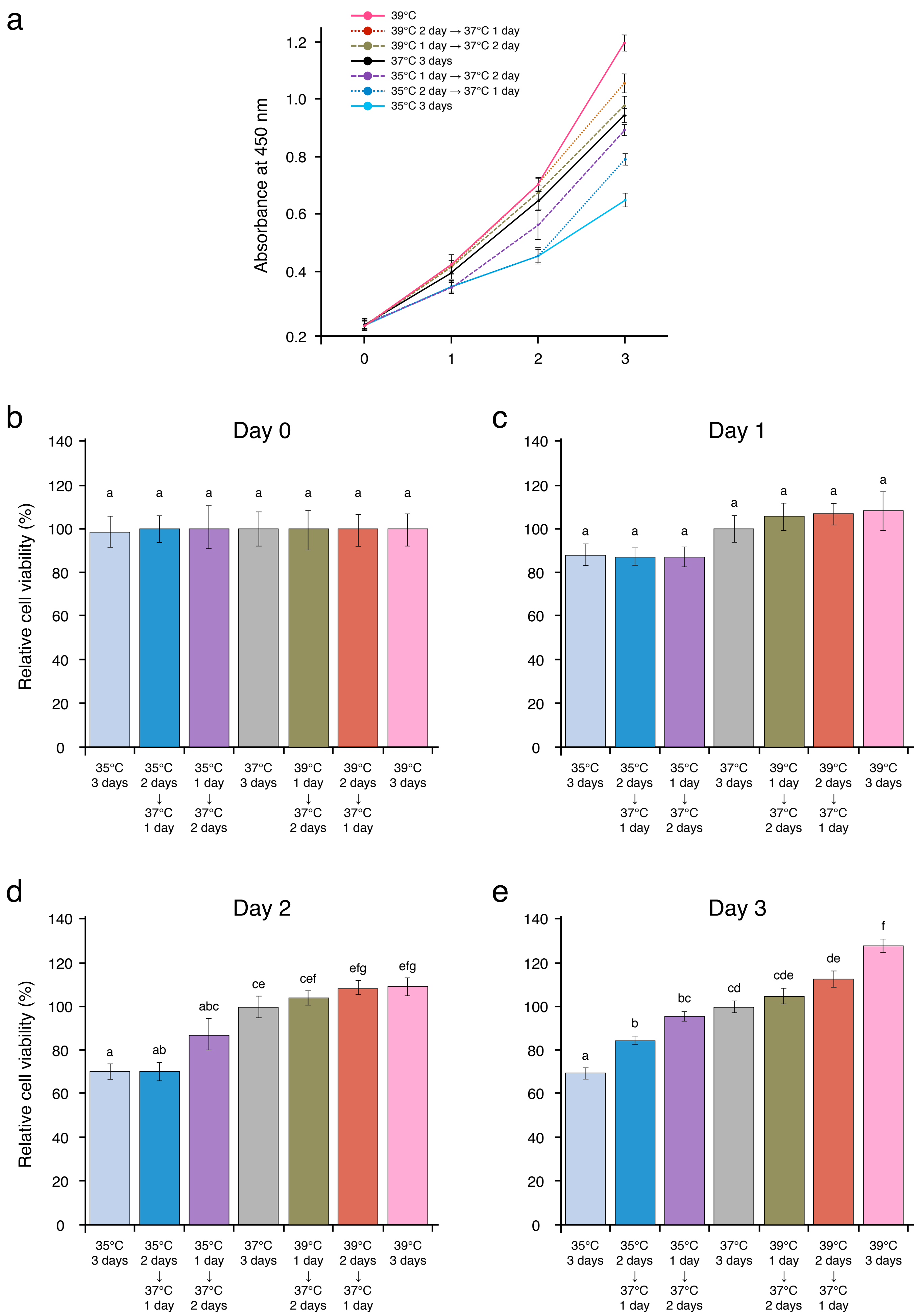

a
39°C
39°C 2 day → 37°C 1 day
39°C 1 day → 37°C 2 day
37°C 3 days
35°C 1 day → 37°C 2 day
35°C 2 day → 37°C 1 day
35°C 3 days
Absorbance at 450 nm
1.2
1.0
0.8
0.6
0.4
0.2
0
1
2
3
b
Day 0
c
Day 1
d
Day 2
e
Day 3
Relative cell viability (%)
140
120
100
80
60
40
20
0
a
ab
abc
ce
cef
efg
b
bc
cd
cde
de
f
35°C 3 days
35°C 2 days ↓ 37°C 1 day
35°C 1 day ↓ 37°C 2 days
37°C 3 days
39°C 1 day ↓ 37°C 2 days
39°C 2 days ↓ 37°C 1 day
39°C 3 days

Figure S5. Effects of temperature shift on the proliferation of breast cancer cells
Effect of temperature shift on the proliferation of basal-type breast cancer cells (MDA-MB-231-luc-D3H2LN cells) (mean ± SEM). The culture temperature was changed at various times during culture. Cell viability was measured at 0, 1, 2, and 3 days after changing the culture temperature (**a**). Cell proliferation was normalized to that of 37°C on day 0 (**b**), day 1 (**c**), day 2 (**d**), or day 3 (**d**). Different letters indicate significant differences at $P < 0.05$. Number of data points per sample was four.

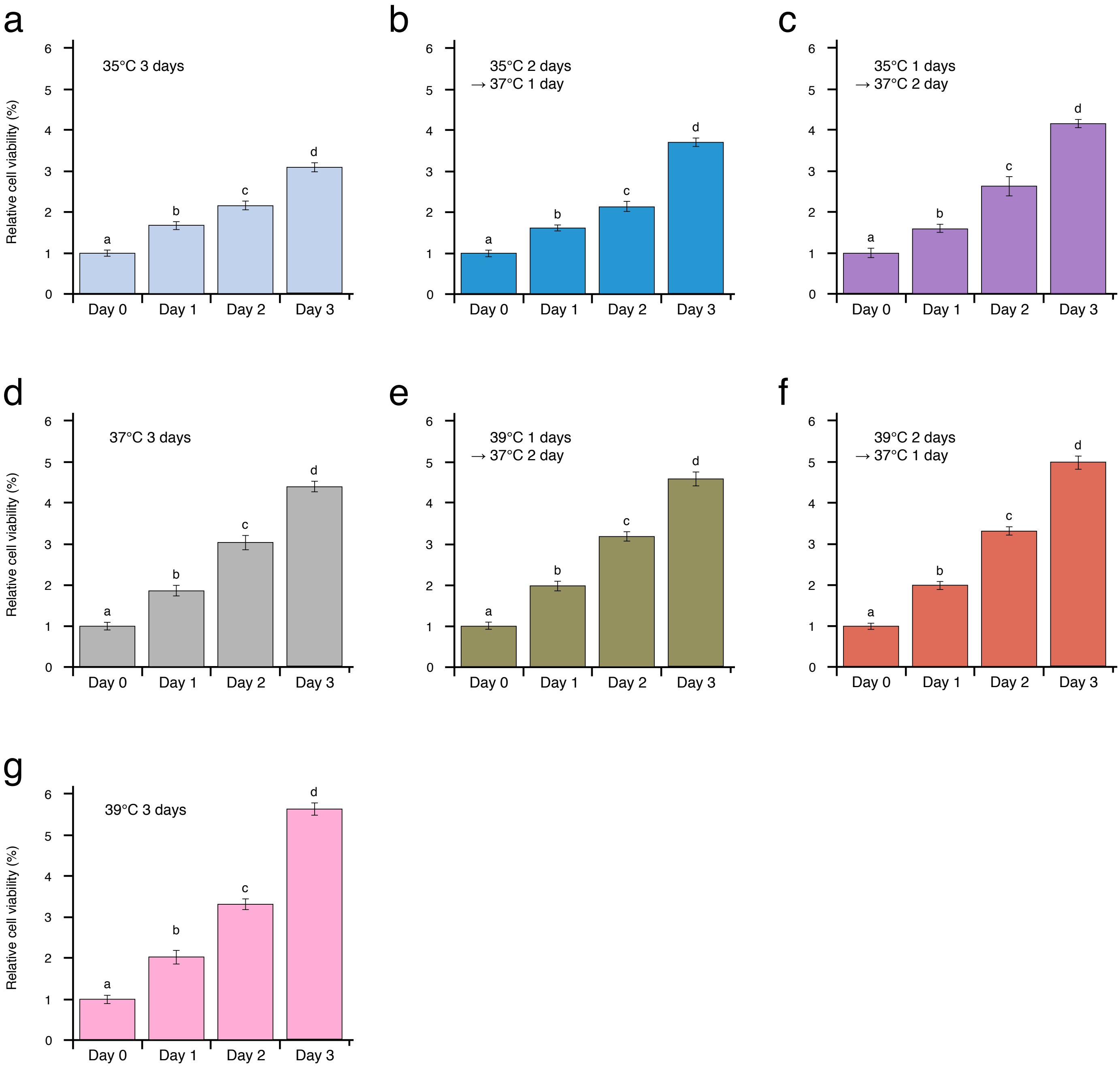

Figure S6. Effects of temperature shift on cell proliferation

(**a–g**) Effect of temperature on the proliferation of basal-type breast cancer cells (MDA-MB-231-luc-D3H2LN cells) (mean ± SEM). The culture temperature was changed at various times during culture (**b**, **c**, **e**, **f**). Cell proliferation was normalized to that of each temperature condition on day 0. Different letters indicate significant differences at $P < 0.05$. Number of data points per sample was four.

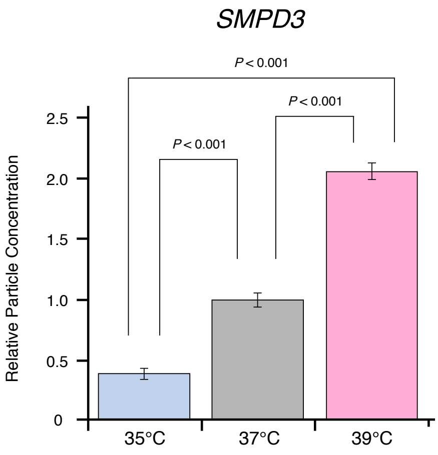

Figure S7. Expression level of *SMPD3* in response to temperature changes
The expression levels were normalized to those at 37°C (mean ± SEM). Number of data points per sample was six.

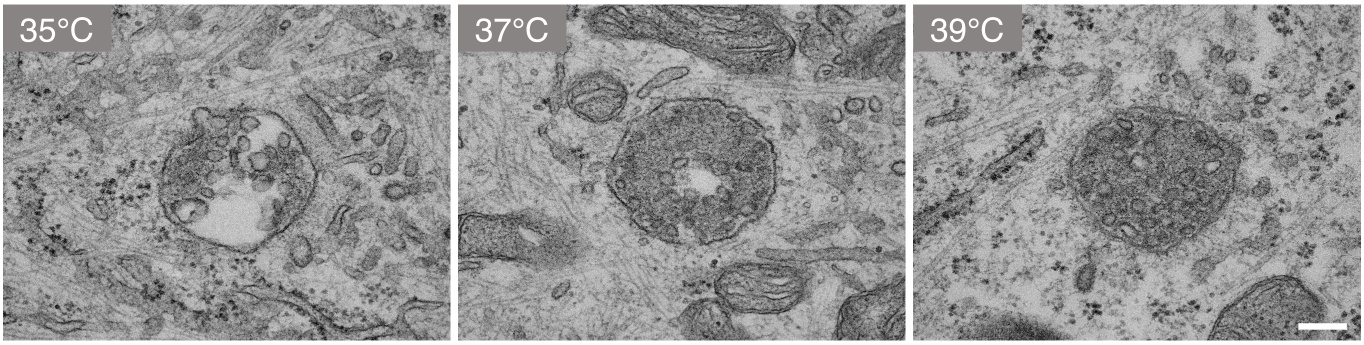

Figure S8. TEM analysis of exosomes within multivesicular bodies
Representative images of exosomes within multivesicular bodies present in MDA-MB-231 cells cultured at 35°C, 37°C, or 39°C. Scale bar represents 200 nm.

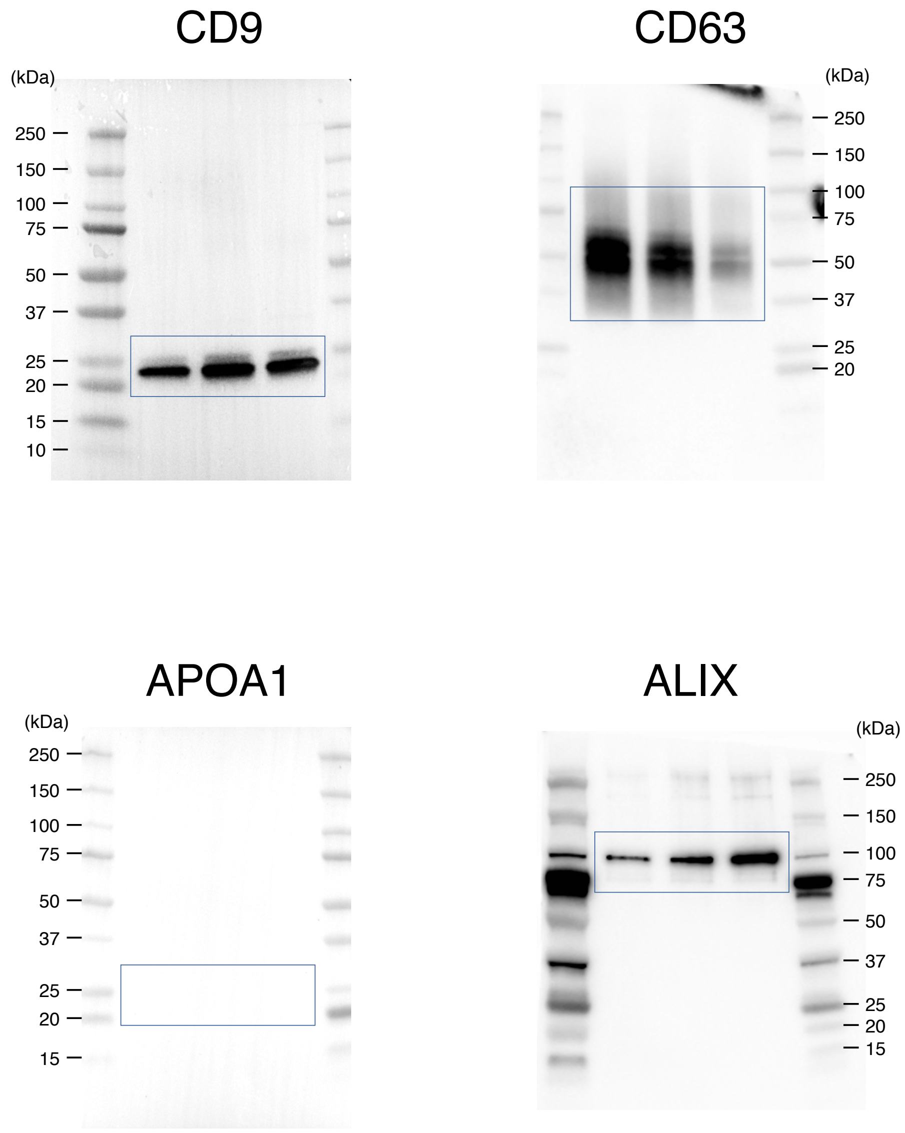

Figure S9. Full scan of the western blots shown in Figure 5(c)

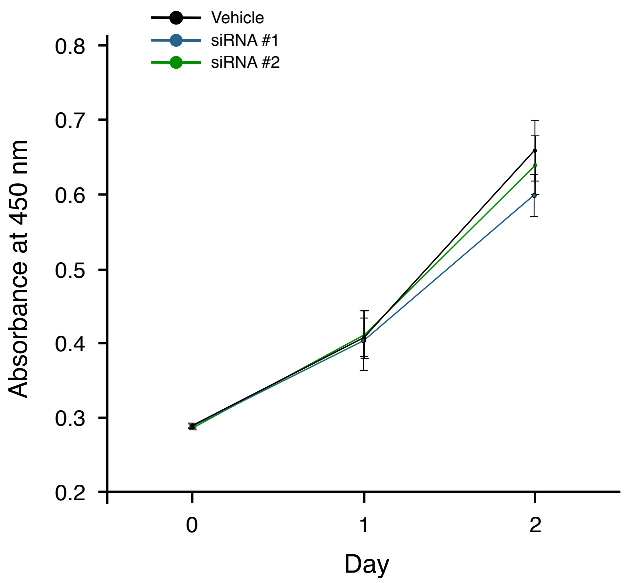

Figure S10. Effect of *LDLR* siRNAs on the proliferation of breast cancer cells

Cell Viability was measured at 0, 1, and 2 days after siRNA transfection (mean ± SEM). Number of data points per sample was five.

a

Number of cells

	cells (x 10^6)
Vehicle	1.94 ± 0.13
siRNA #1	1.78 ± 0.16
siRNA #2	1.82 ± 0.10

mean ± SEM

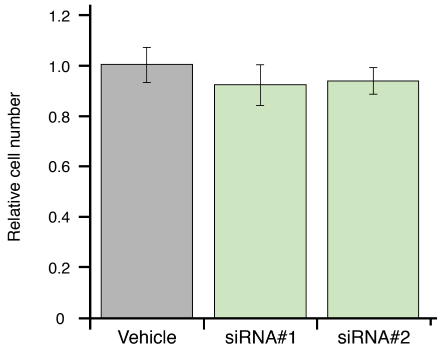

b

Particle concentration

	particles / ml (x 10^9)
Vehicle	3.25 ± 0.40
siRNA #1	1.84 ± 0.28
siRNA #2	1.73 ± 0.21

mean ± SEM

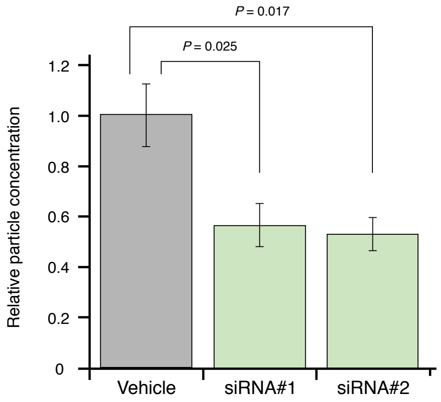

c

Particle concentration

	particles / cell (x 10^3)
Vehicle	9.12 ± 0.56
siRNA #1	5.62 ± 0.53
siRNA #2	5.18 ± 0.44

mean ± SEM

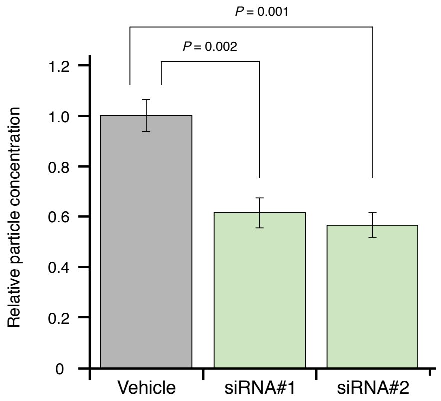

Figure S11. Effects of temperature on exosome secretion in breast cancer
(**a**) Number of cells at the time of exosome isolation. *LDLR* knockdown cells were cultured at 39°C for only 1 day after replacement of fresh medium. Left: number of cells at each temperature. Right: cell numbers normalized to those of vehicle treatment. Number of data points per sample was four.
(**b**) Particle concentration of exosomes from *LDLR* knockdown cells cultured at 39°C (mean ± SEM). Left: particle concentration of exosomes (particles/ml). Right: particle concentration of exosomes normalized to that of vehicle treatment. Number of data points per sample was four.
(**c**) Particle concentration of exosomes from *LDLR* knockdown cells cultured at 39°C (mean ± SEM). Left: particle concentration of exosomes (particles/cell). Right: particle concentration of exosomes normalized to that of vehicle treatment. Number of data points per sample was four.

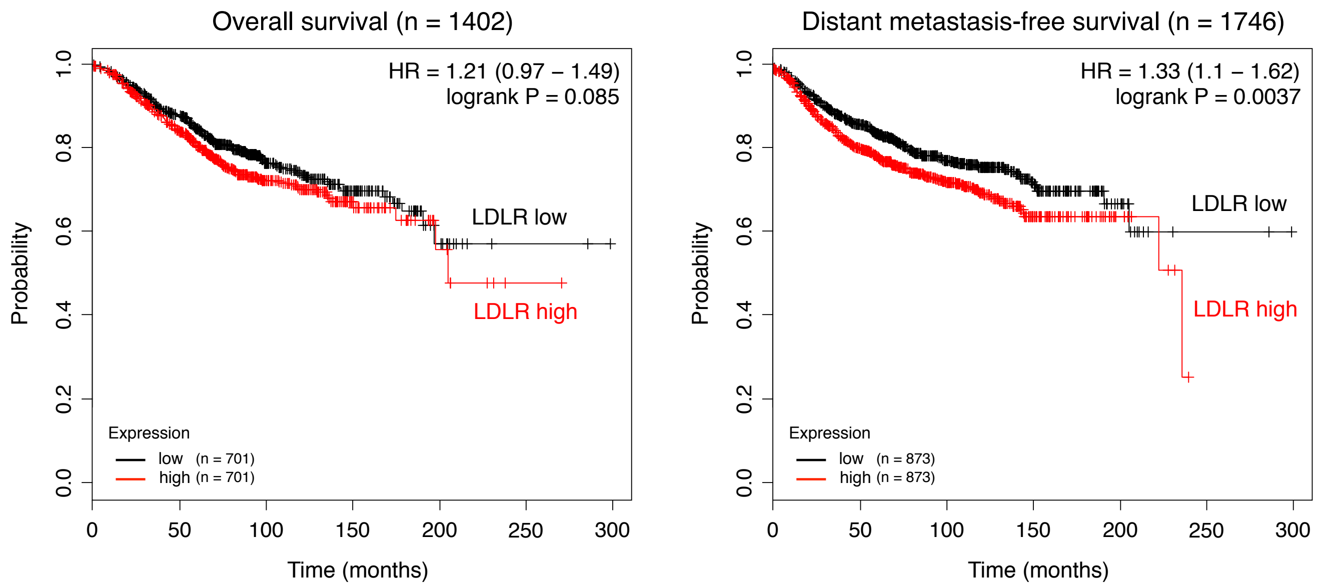

Figure S12. Clinical significance of *LDLR* in breast cancer.

Left: Kaplan-Meier analysis of correlation between overall survival of breast cancer patients with high (above median value, n = 701) and low (below median value, n = 701) *LDLR* levels.

Right: Kaplan-Meier analysis of correlation between distant-free metastasis survival of breast cancer patients with high (above median value, n = 873) and low (below median value, n = 873) *LDLR* levels. *P* value was determined by log-rank test. HR: hazard ratio.
